# African strains of Zika virus resist ISG-mediated restriction

**DOI:** 10.1371/journal.pntd.0013326

**Published:** 2025-07-14

**Authors:** Inès Bribes, Jim Zoladek, Marion Cannac, Sara Salinas, Sam J. Wilson, Sébastien Nisole

**Affiliations:** 1 Institut de Recherche en Infectiologie de Montpellier (IRIM), Université de Montpellier, CNRS, Montpellier, France; 2 Pathogenesis and Control of Chronic and Emerging Infections (PCCEI), INSERM, Etablissement Français du Sang, Université de Montpellier, Montpellier, France; 3 Department of Medicine, Cambridge Institute of Therapeutic Immunology & Infectious Disease (CITIID), Jeffrey Cheah Biomedical Centre, University of Cambridge, Cambridge, United Kingdom; The University of the West Indies, JAMAICA

## Abstract

Zika virus (ZIKV) is a neurotropic *Orthoflavivirus* transmitted by mosquito vectors, which has evolved into two lineages, namely African and Asian. ZIKV from the Asian lineage has been responsible for epidemics in the Pacific and the Americas, the largest of which occurred in Brazil in 2015 and was associated with severe neurological disorders, including cases of microcephaly and other congenital fetal malformations. Although never implicated in human epidemics, African strains exhibit faster replication, higher virus production, and greater virulence in animal models compared to their Asian counterparts. A key feature that may account for the better fitness of African ZIKV strains compared to Asian ones is the fact that they are more resistant to interferon (IFN). IFN response is a major host defense mechanism against viral infections, which culminates in the induction of hundreds of IFN-induced genes (ISGs) whose products inhibit viral replication. By screening an array of ISGs known for their antiviral activity, we show that African ZIKV strains are globally more resistant than their Asian counterparts to ISG-mediated restriction. In particular, SHFL, RTP4 and IFI6, which were the three most active ISGs against Asian viruses, had little or no effect on the replication of African ZIKV strains. These observations therefore suggest that if African strains are more resistant to the antiviral effect of IFN than Asian strains, this is not because they have greater capacity to inhibit IFN signaling, but rather because they are able to escape ISG-mediated restriction. Our results provide an explanation as to why viruses of African origin spread more rapidly and efficiently *in vitro* than their Asian counterparts as repeatedly demonstrated. However, it remains unclear why, despite their greater virulence and resistance to cellular antiviral defenses, ZIKV strains of the African lineage have never been identified in large-scale epidemics.

## Author summary

Interferons (IFNs) are secreted antiviral cytokines, which commonly exert their activity by inducing an antiviral state, conferred by antiviral factors encoded by IFN-stimulated genes (ISGs). Collectively, ISGs can inhibit the replication of all known viruses, although some have developed resistance mechanisms. In this study, we compared the sensitivity to ISGs of different strains of Zika virus (ZIKV), a neurotropic arbovirus that exists as two distinct lineages, African and Asian. The Asian lineage of ZIKV has been responsible for major epidemics associated with severe neurological and congenital disorders, while the African lineage of ZIKV has not been reported to be responsible for epidemics or severe disease so far. In this study, we demonstrate that African ZIKV strains are more resistant than their Asian counterparts to the antiviral activity of IFN, and that ISGs that inhibit replication of Asian viruses have little or no effect on strains of African origin. While our results explain why African ZIKV strains propagate better than Asian ones, as demonstrated in various cellular and animal models, they do not provide an explanation for the higher pathogenicity and epidemic propensity of the Asian lineage.

## Introduction

Zika virus (ZIKV) is a mosquito-borne virus belonging to the *Orthoflavivirus* genus in the *Flaviviridae* family, hereafter referred to as flaviviruses. Following its first isolation in a rhesus macaque monkey in Uganda in 1947 [1], ZIKV remained largely ignored for half a century, during which time it was responsible for a few sporadic human cases in Africa and Asia, associated with mild febrile symptoms.

The first documented human outbreak of ZIKV occurred in 2007, on the island of Yap in Micronesia [2]. In 2013, another, much larger epidemic occurred in French Polynesia, associated for the first time with neurological complications, including Guillain-Barré syndrome in some patients [3]. Following these epidemics, the geographical distribution of ZIKV has expanded considerably, mainly in the Americas and the Western Pacific. From 2015, a ZIKV epidemic of unprecedented scale occurred in Brazil and spread rapidly across the Americas and the Caribbean [4,5]. This epidemic, which affected hundreds of thousands of people, was marked by the incidence of severe neurological disorders, including congenital Zika syndrome, which led the World Health Organization to declare ZIKV a Public Health Emergency of International Concern [4,6,7]. This caused a major transition for ZIKV, from a relatively neglected virus causing mild sporadic infections to a highly pathogenic neurotropic virus with pandemic potential. The virus remains under close international surveillance by health authorities, as it continues to circulate in Asia and Africa.

Phylogenetic analyses have identified two major ZIKV lineages, known as the African and Asian lineages. Strikingly, all human epidemics and cases of congenital and neurological disorders to date have been caused by ZIKV strains belonging to the Asian lineage [8]. This raised the question of whether Asian ZIKV strains were more virulent than African ones. However, a number of *in vitro* and *in vivo* studies have pointed out that African-lineage strains are in fact more virulent and transmissible than their Asian counterparts. Indeed, African ZIKV strains replicate faster, reach higher titers, and are more cytopathic in several cell types [9–18]. They are also more neurovirulent in adult mice and embryos [16,17,19–27], and more efficiently transmitted by mosquitoes [27–32].

These observations therefore suggest that the emergence of Asian ZIKV strains is not due to their higher virulence, but rather to their lower replicative capacity, lower propensity to induce cell death and lower neurovirulence, thus favoring persistent infections [8,33].

To replicate and spread within a cell culture or a living organism, viruses must either avoid, or counteract, the interferon response, the mainstay of innate antiviral immunity [34]. Cells can detect viral components through cell sensors known as pattern-recognition receptors (PRRs), triggering the synthesis of type I interferon (IFN-I). The main PRRs involved in flavivirus sensing are the dsRNA sensors Toll-like receptor 3 (TLR3), retinoic acid-inducible gene I (RIG-I) and melanoma differentiation-associated protein 5 (MDA5) [35]. Once secreted, IFNs activate the Janus kinase/signal transducer and activator of transcription (JAK/STAT) pathway in surrounding cells, triggering the expression of hundreds of IFN-stimulated genes (ISGs) that inhibit viral replication and limit viral spread [36,37]. In the case of flaviviruses, including ZIKV, the main antiviral ISGs include IFI6, RTP4, ISG20, SHFL, Viperin and SLFN11 [38–46].

However, although it has previously been shown that viruses of the African lineage are more resistant to IFN-I than strains belonging to the Asian lineage [18,47], it is not known whether this resistance is due to their greater ability to inhibit IFN signaling or to their greater resistance to the antiviral activity of ISGs. In this study, we carried out a comparative analysis of ZIKV strains of the Asian and African lineage to evaluate their sensitivity to IFN-I and ISGs. First, we confirm previous findings that African ZIKV strains are less sensitive to the antiviral effects of IFN-I than Asian strains. Furthermore, using an array of antiviral ISGs, we demonstrate that while exogenous expression of specific ISGs efficiently inhibited the replication of Asian ZIKV strains, this was not the case for African strains, which were largely resistant. Our results therefore suggest that African strains of ZIKV are more resistant to IFN-I treatment than their Asian counterparts because they can resist ISG-mediated antiviral restriction. Furthermore, our findings provide the molecular basis for why African ZIKV strains replicate faster, reach higher titers, and induce more cell death than Asian strains, as described in various cellular models [9–18].

## Methods

### Cells

Vero E6 (CRL-1586), A549 (CCL-185), HEK 293T (CRL-11268), and C6/36 cells (CRL-1660) were purchased from the American Type Culture Collection (ATCC). All cells, except C6/36, were cultured in high-glucose Dulbecco′s modified Eagle′s medium (DMEM, Gibco) supplemented with 10% fetal bovine serum (FBS, Serana), and 1% Penicillin/Streptomycin (Gibco), and were kept at 37 °C with 5% CO_2_. C6/36 cells were cultured in Leibovitz’s L15 medium (Gibco) supplemented with 10% FBS, 1% Penicillin/Streptomycin, 1% tryptose phosphate broth (Gibco), and 1% nonessential amino acids (Gibco), and were kept at 28 °C with 5% CO_2_.

Buffy coats from healthy donors were obtained from the Etablissement Français du Sang (EFS, Montpellier, France). Human peripheral blood mononuclear cells (PBMCs) were isolated by density gradient centrifugation using Lymphoprep medium (STEMCELL Technologies). Cells were cultured in RPMI 1640 medium (Gibco) supplemented with 10% FBS and 1% penicillin-streptomycin at 37 °C in a 5% CO₂ atmosphere. Monocytes were isolated from PBMCs by plastic adhesion for 45 minutes, and purity was assessed by surface labeling with anti-CD3 and anti-CD14 antibodies (BioLegend), followed by flow cytometry analysis using a Fortessa LSR (BD Biosciences, USA). Data were analyzed with FlowJo (S1 Fig).

### Viruses and infections

Asian Zika virus strains H/PF/2013 and MRS_OPY_Martinique_PaRi_2015 were originally isolated from human sera in French Polynesia (2013) and Martinique (2015), respectively, and obtained from the European Virus Archive GLOBAL (EVAg). The historical African strain ZIKV-#976-Uganda-1947, isolated from a rhesus macaque in Uganda (1947), was also obtained from the EVAg. Another African strain, ArB41644, was isolated mosquitoes captured in Central African Republic in 1989 by the Institut Pasteur Dakar (Senegal), and was kindly provided by the French National Reference Center for Arboviruses (NRC). All viruses had low-passage history (<5 passages on Vero E6 cells). The amino acid sequence identity of the polyprotein encoded by the four ZIKV strains is presented in Table 1.

**Table 1:** Percentage of amino-acid identity in the polyprotein sequence of ZIKV strains. The polyprotein sequences of the different viral strains were compared, and the percentage of sequence identity was calculated. A color scale was applied, ranging from yellow (representing the lowest identity) to green (representing the highest identity).

	Martinique	Polynesia	Uganda	C.A.R.
Martinique	100	99.85	96.84	96.99
Polynesia	99.85	100	96.96	97.11
Uganda	96.84	96.96	100	98.83
C.A.R.	96.99	97.11	98.83	100

Viruses were first amplified on Vero E6 cells, followed by a passage on C6/36 cells. Viral stocks were produced by infecting 80% confluent cells for 2 h, after which the inoculum was removed and replaced with DMEM containing 2% FBS. Supernatants were collected at day 5 for the first passage in Vero E6 cells. In C6/36 cells, supernatants were collected daily from day 3 to day 10, and the supernatant with the highest titer, as defined by flow cytometry, was stored for each strain. Viral titers were determined by TCID_50_ using the Spearman–Kärber method and by quantification of genome equivalents in the supernatant by RT-qPCR.

A549 cells were infected by incubating them with a low volume of virus diluted in serum-free DMEM for 2 hours, using a multiplicity of infection (MOI) appropriate to each experiment. Following incubation, the inoculum was removed and the cells were maintained in DMEM supplemented with 2% FBS. Primary human monocytes were infected by incubating the cells with the appropriate viral dilution in RPMI supplemented with 2% FBS, followed by spinoculation for 10 minutes at 1,000 × g. Eight hours post-infection, the viral inoculum was removed, cells were washed with PBS, and fresh RPMI containing 2% FBS was added. The cells were then incubated for 48 hours at 37 °C.

### Antibodies and reagents

The primary antibodies used were rabbit anti-SHFL (27865-1-AP, Proteintech), rabbit anti-IFI6 (A6157, ABclonal), rabbit anti-RTP4 (NBP1-56448, Bio-Techne), rabbit anti-IFIT2 (12604-1-AP, Proteintech), mouse anti-β-actin (A1978, Merck), mouse anti-flavivirus envelope (NBP2-52666, Novus Biologicals). Secondary antibodies were goat anti-mouse or anti-rabbit HRP conjugates (NA934V, GE Healthcare) and goat anti-mouse AF488 (A11001, Merck). Monocyte phenotyping was carried out by flow cytometry using antibodies anti-CD14-PerCP/Cy5.5 (325621, Biolegend) and anti-CD3-BV421 (300433, Biolegend). To induce ISG expression, cells were treated with Universal type I IFN (PBL Assay Science) at 10, 100 or 1000 U/mL for 16 h. Cell viability was assessed using the CellTiter-Glo Luminescent Cell Viability Assay (Promega), according to the manufacturer’s instructions.

### Western blot

Confluent cell monolayers were washed with PBS, and either lysed with ice-cold RIPA buffer (25 mM Tris-HCl [pH 7.5], 150 mM NaCl, 0.5% sodium deoxycholate, 1% NP-40, 1 mM EDTA, 2.5 mM sodium pyrophosphate, 1 mM sodium orthovanadate, and 1 mM β-Glycerophosphate) for 15 min and centrifuged at 15,000 × g for 10 min to remove insoluble debris; or, for IFI6 detection, lysed with room-temperature 2% SDS in water for 5 min and then sonicated (Fisherbrand 505 Sonicator with Probe) for two cycles of 5 sec.

Samples were then heat-denatured at 95 °C for 10 min in 4X Laemmli buffer (250 mM Tris-HCl [pH 7], 8% sodium dodecyl sulfate [SDS], 40% glycerol, 10% β-mercaptoethanol, and 0.005% bromophenol blue). Sample then underwent SDS polyacrylamide gel electrophoresis (SureCast Gel Handcast System, Thermo Fisher Scientific), followed by transfer onto a 0.45 µm nitrocellulose membrane (Amersham) or, for the detection of IFI6, onto a 0.45 µm polyvinylidene difluoride membrane (Immobilon-FL), activated in 100% methanol beforehand. Membranes were saturated in PBS, 0.05% Tween 20 (PBST) with 10% fat-free milk for 30 min. Primary and secondary antibodies were diluted in PBST, 1% BSA. Primary antibody was incubated either for 1 h at room-temperature or 16 h at 4°C. Secondary antibody was incubated for 1 h at room-temperature. Protein visualization was achieved by HRP activity (Immobilon Forte Western HRP substrate, Merck) on a ChemiDoc imaging system (Bio-Rad). Images were analyzed with ImageJ.

### Real-time quantitative RT-PCR (RT-qPCR)

Total RNAs were extracted using the NucleoSpin RNA plus kit (Macherey-Nagel) following the manufacturer′s instructions. RNA concentration and purity were evaluated by spectrophotometry (NanoDrop 2000c, Thermo Fisher Scientific). A maximum of 500 ng of RNA were reverse transcribed with both oligo dT and random primers using a PrimeScript RTReagent Kit (Perfect Real Time, Takara Bio Inc.) in a 10 µL reaction. Real-time PCR reactions were performed in triplicate using Master Mix PowerUp SYBR Green (Thermo Fisher Scientific) on an Applied Biosystems ViiA 7 Real-Time PCR System (Thermo Fisher Scientific). ZIKV amplification was performed using the following primers: Forward: 5’-GACTGGGTTCCAACTGGGAG-3’, Reverse: 5’-CCACACTCTGTTCCACACCA-3’, and the following program: 2 min at 50 °C, 2 min at 95 °C, and 45 cycles of 15 s at 95 °C followed by 1 min at 60 °C. A melting curve analysis was also performed. Absolute copy number of ZIKV RNA copies was determined by standard curve interpolation using a plasmid into which ZIKV amplicon has been cloned (pCR2.1-TOPO cloning kit, Thermo Fisher Scientific). For the quantification of ISG transcripts in Vero E6 and A549 cells, real-time PCR reactions were performed in duplicate using Takyon ROX SYBR MasterMix blue dTTP (Eurogentec) on an Applied Biosystems QuantStudio 5 (Thermo Fisher Scientific). Transcripts were quantified with the following program: 3 min at 95°C, followed by 40 cycles of 15 s at 95°C, 20 s at 60°Cand 20 s at 72°C. Values for each transcript were normalized to expression levels of RPL13A (60S ribosomal protein L13a), using the 2^-ΔΔCt^ method. Primers used for quantification of transcripts are indicated within Table 2.

**Table 2:** Primers used in RT-qPCR analyses of ISG transcripts.

Species	Target transcript	F/R	Sequence (5’- > 3’)
*Homo sapiens* (A549 cells)	RPL13A	F	AACAGCTCATGAGGCTACGG
		R	TGGGTCTTGAGGACCTCTGT
	Mx2	F	GAAAAGCGTCATGAATGTGGT
		R	TCAGCCTGTTTGTGATCTCCT
	IFIT1	F	ATGCGATCTCTGCCTATCGC
		R	CCTGCCTTAGGGGAAGCAAA
	IFITM1	F	AGGAAGATGGTTGGCGACG
		R	GCCGAATACCAGTAACAGGATGA
	SHFL	F	GTATCCTCCAAGAAGGCGGG
		R	TTGCTTTACCCCGTACACGA
	IFI6	F	GGGTGGAGGCAGGTAAGAAA
		R	GTCAGGGCCTTCCAGAACC
	RTP4	F	CAGGGTCAGGTGCGTATGAG
		R	ATATGCTGCACCAGGTTGCT
	ISG15	F	CAGCGAACTCATCTTTGCCAG
		R	GACACCTGGAATTCGTTGCC
	ISG20	F	GAGCGCCTCCTACACAAGAG
		R	TAGAGCTCCATCGTTGCCCT
*Chlorocebus sabaeus* (Vero E6 cells)	RPL13A	F	CCACCGCCCTATGACAAGAA
		R	TACTTCCAGCCAACCTCGTG
	Mx2	F	CAAACTGGCAGGGGGTAGAG
		R	TCGTGTTTCCTGGTAGTGGC
	IFIT1	F	AGGAAACACCCACTTCGGTC
		R	TAGGCTGCCCTTTTGTAGCC
	IFITM1	F	ACATCTCGGCCCTGATTGTG
		R	GGTAGATTGCCACAGAGCCA
	SHFL	F	TGCCAGCTGACAAGATGTGG
		R	CGTGTTGGATACACGGGGAA
	IFI6	F	CTGCGATCATGAATGGGGGT
		R	CCCAGCAGGGCACCTATTTTA
	RTP4	F	CTGGTTCCGGTGTTCTTCCT
		R	AAGAGCCTCATACGCACCTG
	ISG15	F	GTGGTGGACAGATGCGATGA
		R	GTACCTCGTAGGTGCTGCTC
	ISG20	F	CTCCTGCACAAGAGCATCCA
		R	TGGTAGAGCTCCATCGTTGC

### ISG challenge

ISG overexpression was performed using lentiviral constructions from the SCRPSY library (GenBank accession no. KT368137) each co-expressing an ISG (see Table 3) with red fluorescent protein (TagRFP) and puromycin resistance, as described previously [42,43,48,49]. Briefly, ISG-encoding lentiviral vectors were generated by co-transfecting SCRPSY-based ISG expression vectors with VSV-G (pMD2.G, Addgene accession no. 12259), and HIV-1 gag-pol (pCMVR8.74, Addgene accession no. 22036) in HEK 293T cells using polyethylenimine (PEI, Polysciences). Supernatants containing the lentiviral vectors were collected 48 h post-transfection and kept at -80 °C. Lentivector titers were determined by flow cytometry.

**Table 3:** List of human and simian ISGs included in restriction assays.

Species	Gene symbol	Alias
Human	BRIP1	BACH1, FANCJ
	EIF2AK2	PKR
	GRIP2	
	IFI44L	
	IFI6	
	IFIT1	IFI56
	IFIT2	IFI54
	IFITM1	
	IFITM2	
	IFITM3	
	ISG15	
	ISG20	
	LY6E	
	MS4A4A	
	MX2	
	PAK3	
	PARP10	
	PARP12	
	PML	TRIM19
	RSAD2	Viperin
	RTP4	
	SAMHD1	
	SHFL	C19orf66, RyDEN
	SIRPA	PTPNS1
	THEMIS2	
	TRIM5	
	TRIM69	RNF36
Simian	IFI6	
	RTP4	

Vero E6 cells were transduced with each ISG-encoding lentivector at 1 × TCID_50_, resulting in TagRFP (and thus ISG) expression in approximately 50% of the cells after 72 h. Following transduction, cells were infected at 1 × TCID_50_ for 72 h. In this specific context, the infection inoculum was maintained on the cells for the duration of the experiment. Viral replication was assessed in TagRFP-negative and TagRFP-positive cells by flow cytometry, as previously described [42,43,48,49].

### Statistical analysis

Statistical analyses were performed using GraphPad Prism version 10 (GraphPad Software). Data distribution was assessed for normality prior to analysis, and appropriate statistical tests were selected accordingly. Depending on the distribution and experimental design, either parametric (one-way or two-way ANOVA) or non-parametric (Mann–Whitney U test) tests were applied, followed by the relevant multiple comparison procedures (Tukey’s, Fisher’s LSD, or Sidak’s tests). The specific test used for each dataset is indicated in the corresponding figure legends. All tests were two-tailed, and p-values less than 0.05 were considered statistically significant.

## Results

We carried out a comparative analysis of the sensitivity of ZIKV lineages to IFN and ISGs on four strains. Since the prototype African strain MR766 has undergone extensive passage history in suckling mouse brains [50], two low-passaged African strains were selected for the study, namely an isolate obtained from mosquitoes in Central African Republic (C.A.R.) in 1989 (ArB41644) and the historical strain isolated from a monkey in Uganda in 1947 (#976-Uganda-1947). Two Asian strains were also included: the ZIKV strains H/PF/2013 and MRS_OPY_Martinique_PaRi_2015, which were isolated from human sera in French Polynesia in 2013 and Martinique in 2015, respectively. In the rest of the manuscript, ZIKV strains are referred to according to their origin (*i.e.,* C.A.R. and Uganda for African ZIKV strains, and Martinique and Polynesia for Asian ZIKV strains).

**Fig 1:**
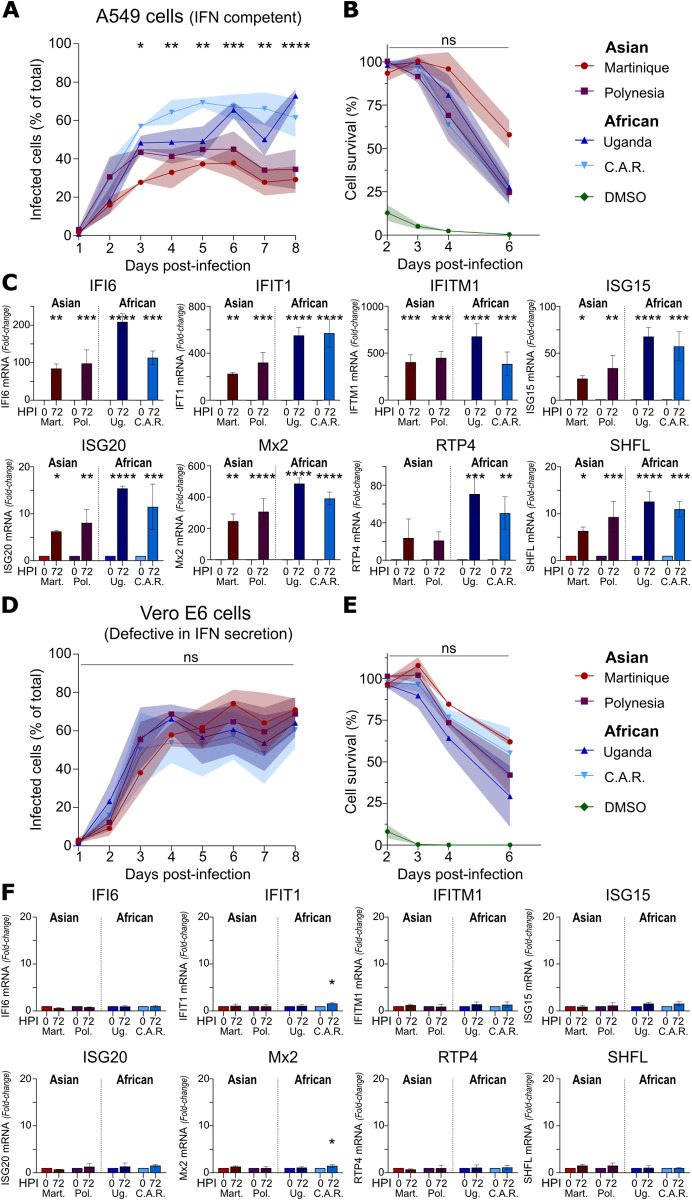
Replication kinetics of African and Asian ZIKV strains. (A–C) Viral infection dynamics in A549 cells (IFN-competent). (A) Replication kinetics: Cells were infected with each ZIKV strain at a MOI of 0.01, and the percentage of infected cells was quantified daily for 8 days by flow cytometry. (B) Cell viability: Viability was measured on days 2, 3, 4, and 6 post-infection using a luminescent assay and expressed as a percentage relative to uninfected controls. (C) Interferon-stimulated gene (ISG) induction: ISG expression was assessed by RT-qPCR at 0 and 72 hours post-infection, with fold changes calculated relative to baseline (0 hpi). (D–F) Viral infection dynamics in Vero E6 cells (IFN-deficient). (D) Replication kinetics: Cells were infected at a MOI of 0.01, and the percentage of infected cells was measured daily over 8 days by flow cytometry. (E) Cell viability post-infection, measured and analyzed as described in panel B. (F) ISG induction, assessed and analyzed as in panel C. For all panels, data are presented as mean ± SD (N = 3). Statistical comparisons of infection rates (panels A and D) and cell viability (panels B and E) between Asian and African strains were performed using two-way ANOVA followed by Fisher’s LSD post hoc test. For ISG induction (panels C, F), comparisons between 0 and 72 hpi for each virus were conducted using two-way ANOVA with Sidak’s multiple comparisons test. Statistical significance: ns, p > 0.05; *, p < 0.05; **, p < 0.01; ***, p < 0.001; ****, p < 0.0001.

We first assessed the replication kinetics of the four ZIKV strains in human A549 and simian Vero E6 cells. To this end, cells were infected at a multiplicity of infection (MOI) of 0.01, and the proportion of infected cells was monitored over time by flow cytometry following viral envelope protein labeling. In A549 cells, African strains exhibited faster replication and achieved higher titers, with over 60% of cells infected by day 6, compared to less than 40% for the Asian strains (Fig 1A). To rule out differential cytopathic effects as a cause of the observed differences in replication kinetics, we assessed virus-induced cell death up to day 6 post-infection and found it to be significantly similar between African and Asian ZIKV strains (Fig 1B). As expected, ZIKV infection elicited a robust induction of antiviral ISGs in A549 cells, as measured by RT-qPCR at 72 hours post-infection (Fig 1C). In contrast, all four strains displayed similar replication kinetics in Vero E6 cells, (Fig 1D), and virus-induced cell death was also comparable across ZIKV lineages (Fig 1E). Consistent with their known deficiency in IFN secretion upon viral infection [51], Vero E6 cells showed no detectable ISG expression at 72 hours post-infection (Fig 1F). These observations suggest that Asian strains are more susceptible to IFN response compared to African strains as observed in A549 cells, while both lineages replicate at similar rates in IFN-deficient Vero E6 cells.

**Fig 2:**
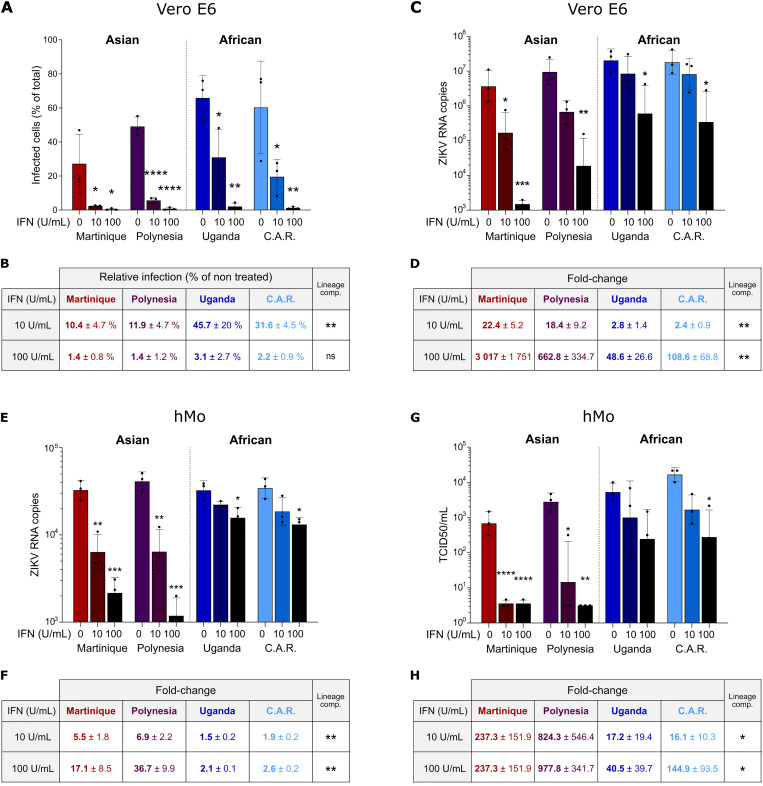
African ZIKV strains exhibit greater resistance to IFN-I treatment compared to Asian strains. (A) IFN-I susceptibility of various ZIKV strains was assessed in Vero E6 cells by flow cytometry. Cells were pre-treated with IFN-I (0, 10, or 100 U/mL) for 16 hours and subsequently infected at MOI 0.1. Infection was measured at 48 h post-infection by flow cytometry. Data are shown as individual replicates with mean ± SD (N = 3). Statistical analysis compares each treatment to the untreated control for each strain (one-way ANOVA with Tukey’s multiple comparisons). (B) Relative infection levels from panel A, normalized to untreated controls. Data are shown as mean ± SD (N = 3). Statistical comparison was performed between Asian and African strains using a Mann–Whitney test. (C) Viral RNA levels were quantified in Vero E6 cells infected with ZIKV strains (normalized to 10⁴ genome equivalents/well) following 16 h IFN-I pre-treatment (0, 10, or 100 U/mL). RNA was extracted at 48 h post-infection. Data are shown as individual replicates with mean ± SD (N = 3). Statistical comparisons were made as in panel A. (D) Fold change in RNA levels from panel C, calculated relative to untreated controls. Data are shown as mean ± SD (N = 3), with statistical comparisons between Asian and African ZIKV strains (Mann–Whitney test). (E) IFN-I susceptibility was assessed in human primary monocytes infected with ZIKV strains (10⁶ genome equivalents/well) following 16 h IFN-I treatment (0, 10, or 100 U/mL). RNA was extracted 48 h post-infection. Data are shown as individual replicates with mean ± SD (N = 3). Data were analyzed by one-way ANOVA with Tukey’s test. (F) Fold change in RNA levels from panel E, relative to untreated controls. Data are presented as mean ± SD (N = 3). Statistical comparison between Asian and African ZIKV strains was performed using an unpaired t-test. (G) Viral titers in supernatants from IFN-I-treated primary monocytes (same setup as panel E) were measured at 48 h post-infection using TCID₅₀ assays on Vero cells. Data are shown as individual replicates with mean ± SD (N = 3). Statistical analysis was conducted as in panel A. (H) Fold change in viral titers from panel G, relative to untreated conditions. Data are presented as mean ± SD (N = 3), with group comparisons between Asian and African ZIKV strains using a Mann–Whitney test. Statistical significance: ns, p > 0.05; *, p < 0.05; **, p < 0.01; ***, p < 0.001; ****, p < 0.0001.

To confirm the greater sensitivity of the two Asian strains to the antiviral activity of IFN compared to African strains, we treated Vero E6 cells with 10 or 100 U/mL of Universal Type I IFN for 16 h prior to infection. After 48 h of infection, the percentage of infected cells was assessed by flow cytometry. While a high IFN concentration (100 U/mL) completely abolished replication of all 4 viral strains, we were able to observe differences in sensitivity of ZIKV strains following treatment with the lower dose (10 U/mL) (Fig 2A), with the Asian strains showing a significantly greater reduction in the percentage of infected cells compared to the African strains (Fig 2B). This result was further validated by quantifying viral RNA levels in infected cells by RT-qPCR (Fig 2C and 2D), and by measuring viral titers by TCID_50_ in the supernatant of infected cells (S2A and S2B Fig).

In order to validate these results in a more physiologically relevant model, we repeated these experiments in human primary monocytes, which are the main targets of ZIKV [52,53]. As in Vero E6 cells, Asian strains exhibited greater susceptibility to IFN treatment than African strains in human monocytes, as demonstrated by RT-qPCR (Fig 2E and 2F) and TCID_50_ assays (Fig 2G and 2H). Our results therefore demonstrate that Asian ZIKV strains are more sensitive to IFN than African strains in both Vero E6 cells and human monocytes, in agreement with previous studies [18,47].

Given that IFN exerts its antiviral activity through the induction of ISG products, the different susceptibility of ZIKV strains to IFN suggests that they may have different susceptibilities to ISGs. To test this hypothesis, we screened a library of 24 human ISGs with known antiviral activity against flaviviruses, including IFI6, ISG20, RTP4, and SHFL (Table 3). The ISGs were expressed using lentiviral vectors (SCRPSY), with each vector expressing an ISG and the TagRFP reporter protein, as previously described [42,43,48,49]. We also included two simian ISGs (IFI6 and RTP4), which are expected to have antiviral activity similar to their human orthologs, and three human ISGs with no known antiviral effect (BRP1, THEMIS2 and GRIP2) which served as negative controls (Table 3) [54].

**Fig 3:**
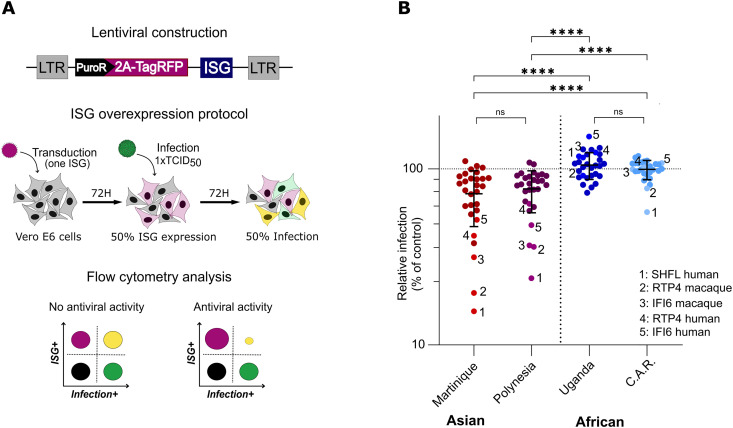
African ZIKV strains are more resistant to a wide-range of ISGs than Asian strains. (A) Schematic representation of the SCRPSY lentiviral construction used and the experimental procedure. ISG sensitivity of Asian and African strains of ZIKV was tested in Vero E6 cells transduced with individual lentivectors, each expressing a single ISG, for 72 h, followed by infection with 1 × TCID_50_ of ZIKV for an additional 72 hours. (B) Relative ZIKV infection (as a percentage of empty vector) in Vero E6 cells was assessed by flow cytometry. Each dot corresponds to the mean of three biological replicates for each ISG. Data are presented as individual ISGs and mean ± SD (one-way ANOVA followed with Tukey’s multiple comparisons test). Statistical significance: ns, p > 0.05; *, p < 0.05; **, p < 0.01; ***, p < 0.001; ****, p < 0.0001.

In order to assess the sensitivity of ZIKV strains to the antiviral activity of ISGs, we overexpressed them in Vero E6 cells to avoid the potentially confounding effects that would otherwise arise from IFN secretion. Vero E6 cells were transduced with individual lentiviral vectors, each expressing a single ISG, for 72 h at a MOI leading to approximately 50% of transduced cells. Following transduction, cells were infected with the different ZIKV strains resulting in around 50% of infected cells (*i.e.,* 1 x TCID_50_) at 72h post-infection (Fig 3A). The viral replication in TagRFP-negative and TagRFP-positive cells was then assessed by flow cytometry, measuring viral envelope protein expression (Fig 3A). The infection rate of each virus in the presence of the individual ISGs was represented as a percentage of infection relative to control cells transduced with the empty vector, determined by flow cytometry (Fig 3B). Strikingly, Asian and African strains of ZIKV showed completely different susceptibilities to ISG overexpression, as illustrated by the contrasting overall patterns of the dot cluster profiles. Indeed, many ISGs inhibited the replication of Asian viruses, resulting in a downward scattering of data points, indicating reduced infection. In line with the literature, the most effective ISGs were SHFL [44,46], RTP4 [40] and IFI6 [38,39]. It is noteworthy that simian SHFL and RTP4 showed the same restriction efficiency as their human orthologs (Fig 3B). In the case of African viruses, on the other hand, the scatterplot was clustered around the control, illustrating a striking overall lack of sensitivity to ISGs. Only the overexpression of human SHFL led to a slight decrease in infection of the ArB41644 virus (noted as C.A.R. in Fig 3B). We observed the same trend when examining the mean fluorescence intensity (MFI) of the ZIKV-E protein in single-infected cells and in ISG-expressing ZIKV-infected cells, rather than the percentage of infected cells (S3 Fig). This further confirmed that ISG expression effectively reduced ZIKV-E protein production in infected cells, with a more pronounced effect on African ZIKV strains compared to Asian strains.

**Fig 4:**
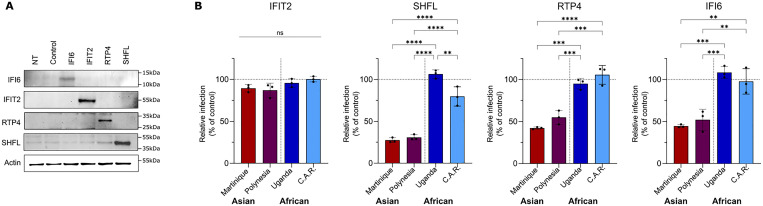
African ZIKV strains are resistant to the antiviral activity of human SHFL, RTP4 and IFI6. (A) Western blot validation of IFI6, IFIT2, RTP4, and SHFL overexpression in Vero E6 cells transduced with lentivectors coding for the corresponding ISG for 72 h. Empty vector was used as a control. (B) Relative ZIKV infection in IFIT2, SHFL, RTP4 and IFI6 overexpressing Vero E6 cells (as a percentage of empty vector). Cells were transduced for 72 h with lentivectors encoding the corresponding ISGs and infected at 1 × TCID_50_ of ZIKV. The percentage of infected cells was assessed after 72 h post-infection by flow cytometry. Data corresponds to the relative infection for each ISG measured as a percentage of the control condition (empty vector) and are represented as individual replicates and mean ± SD (N = 3) (one-way ANOVA followed by Tukey’s multiple comparisons test). Statistical significance: ns, p > 0.05; *, p < 0.05; **, p < 0.01; ***, p < 0.001; ****, p < 0.0001.

To confirm this difference in susceptibility to ISG-mediated restriction between African and Asian ZIKV strains, we conducted three independent experiments focusing on the ISGs that exhibited the strongest inhibition of Asian strains, namely IFI6, RTP4 and SHFL. We also included IFIT2 as a negative control, as this ISG showed no effect on any tested ZIKV (Fig 3B). Vero E6 cells were transduced with the ISG-expressing vectors and ISG expression was validated by Western blot (Fig 4A). Cells were then infected with the different ZIKV strains for 72 h and the infection was assessed by flow cytometry. As expected, IFIT2 overexpression had no effect on the replication of any of the four tested viruses (Fig 4B). In contrast, and in agreement with our ISG screen, the overexpression of SHFL, RTP4 and IFI6 inhibited replication of the Asian ZIKV strains, without affecting that of African strains. Of note, only the African strain ArB41644 (C.A.R.) underwent a slight but significant decrease in infection following SHFL overexpression, while the Uganda-1947 strain was not affected at all. On the other hand, overexpression of IFI6 and RTP4 had no effect on the infection of either ArB41644 or Uganda-1947, while efficiently interfering with that of the two Asian strains (Fig 4B).

Taken together, our results therefore demonstrate that ZIKV strains of the African lineage display better resistance to the antiviral activity of ISGs than their Asian counterparts.

## Discussion

We compared the sensitivity of ZIKV strains derived from Asian and African lineages to IFN-induced antiviral cellular factors. We showed that African strains replicate faster than their Asian counterparts in A549 cells, a human lung-derived cell line that is competent for the IFN response. In contrast, we found no significant difference in replication kinetics between African and Asian strains in Vero E6 cells, which are derived from African green monkey kidney and lack the ability to secrete IFN upon infection [51]. Although the species and tissue differences between A549 and Vero E6 cells could influence viral replication independently of IFN competence, this initial observation suggested that Asian strains might be more susceptible to the IFN response than African strains. By treating Vero E6 cells or primary human monocytes with exogenous IFN-I, we were able to confirm that strains derived from the African lineage were less sensitive to IFN-induced inhibition compared to their Asian counterparts, as previously described [47]. Building on these observations, we next evaluated the sensitivity of selected ZIKV strains to the antiviral activity of ISGs by individually overexpressing them in Vero E6 cells. Consistent with their greater resistance to IFN-I, African ZIKV strains showed little or no impact from the overexpression of antiviral ISGs such as SHFL, RTP4 and IFI6, whereas Asian strains suffered dramatic inhibition of replication. These results suggest that the resistance of African ZIKV strains to the antiviral effects of IFN is not due to a superior ability to inhibit IFN signaling but rather to their ability to evade restriction by antiviral ISGs. Overall, our findings demonstrate that African ZIKV strains are more resistant to IFN-I treatment than their Asian counterparts, due to their ability to evade ISG-mediated antiviral restriction. This resistance offers a molecular basis for the faster replication, higher viral titers, and increased cell death observed with African strains, as compared to Asian strains, across a range of cell types [9–18] and mouse models [16,17,19–27].

However, how African strains manage to escape ISG-mediated restriction remains an open question. It should be noted that SHFL, RTP4 and IFI6, which we identified as inducing the strongest inhibition of Asian ZIKV strains*, i.e.,* SHFL, IFI6 and RTP4, all act at the same stage of the flavivirus replication cycle, namely the replication of viral RNA, which takes place in invaginations of the endoplasmic reticulum. It is conceivable that African ZIKV strains form viral factories more efficiently than their Asian counterparts, or that these factories are less accessible to restriction factors. In line with the latter hypothesis, ZIKV strains of the African lineage have been shown to induce less IFN and at a slower rate than Asian strains [14,18,55], suggesting that their RNA is better shielded from sensor PRRs.

The fact that African ZIKV strains are able to resist the antiviral activity of ISGs could explain why African ZIKV strains replicate faster, at higher titers and induce more cell death than their Asian counterparts, as described in various cell models [9–18]. However, crucial questions remain unanswered. First, why is it that, while African ZIKV viruses have been shown to have better fitness [9–18], increased neurovirulence [16,17,19–27] and even better transmissibility [27–32], only the Asian strains have caused epidemics with dramatic outcomes? Second, why would a highly virulent virus, capable of resisting antiviral cellular factors, evolve to lose this capacity?

There are at least two possible explanations for these apparent contradictions. The first is that a low virulence, slow replication rate and sensitivity to innate immune defenses is likely to favor persistent infections in immune sanctuaries such as the brain, conferring a selective advantage over strains that replicate faster, evade cellular defenses and induce increased cytotoxicity [8,33]. Indeed, a number of studies have shown that Asian-lineage strains can replicate at low level and persist in certain tissues, including the brain, months after initial infection [56,57]. The second hypothesis is that epidemics due to African strains go more easily unnoticed by public health surveillance systems than Asian strains, due to their propensity to cause fetal loss rather than congenital malformations [27]. However, it is unlikely that a single factor accounts for the lack of epidemic potential of African ZIKV strains. Instead, multiple factors, such as environmental conditions or vector-related constraints, likely contribute to limiting their spread [58–61]. Future research on host-virus interactions, transmission dynamics, vector competence, and ecological factors in the different regions where the virus circulates will be needed to better understand the disparities between ZIKV lineages.

## Supporting information

S1 Fig Phenotypic characterization of human primary monocytes. Human monocytes were isolated from peripheral blood mononuclear cells (PBMCs) by plastic adhesion. Following isolation, cells were stained for CD3 and CD14 to assess purity and analyzed by flow cytometry. Representative dot plots from a typical experiment are shown.
(TIF)

S2 Fig Quantification of viral titers of ZIKV strains following IFN treatment in Vero E6 cells. (A) Susceptibility of ZIKV strains to IFN-I was assessed by measuring viral titers in Vero E6 cells. Cells were pre-treated with IFN-I (0, 10, or 100 U/mL) for 16 hours, followed by infection with different ZIKV strains (MOI 0.1). Supernatants were collected at 48 h post-infection, and viral titers were determined by TCID₅₀ assay on Vero cells. Data are shown as individual replicates with mean ± SD (N = 3). Statistical comparisons were made between IFN-treated and untreated conditions for each strain independently using one-way ANOVA with Tukey’s multiple comparisons test. (B) Fold-change in viral titers from panel A, normalized to untreated controls. Data are presented as mean ± SD (N = 3). Statistical comparison between Asian and African strains was performed using a Mann–Whitney test. Statistical significance: *, p < 0.05; **, p < 0.01.
(TIF)

S3 Fig ZIKV infection levels in Vero E6 cells overexpressing individual ISGs, measured by mean fluorescence intensity. Vero E6 cells were transduced with individual interferon-stimulated genes (ISGs) and subsequently infected with ZIKV. Infection was quantified by flow cytometry based on mean fluorescence intensity (MFI), expressed relative to cells transduced with an empty vector control (set to 100%). Each data point represents the mean of three biological replicates per ISG. Data are shown as individual ISGs with mean ± SD. Statistical analysis was performed using one-way ANOVA followed by Tukey’s multiple comparisons test. Statistical significance: ns, p > 0.05; *, p < 0.05; **, p < 0.01; ***, p < 0.001.
(TIF)

S1 Data Raw data for Figs 1A,1B,1C,1D,1E,1F,2A–B,2C–D,2E–F,2G–H,3B,4B,S1 and S3. (XLSX)
