# Supplementary figures and images for: African strains of Zika virus resist ISG-mediated restriction
Source: PLoS Negl Trop Dis. 2025 Jul 14;19(7):e0013326. doi: 10.1371/journal.pntd.0013326 (PMC12270304; doi:10.1371/journal.pntd.0013326)

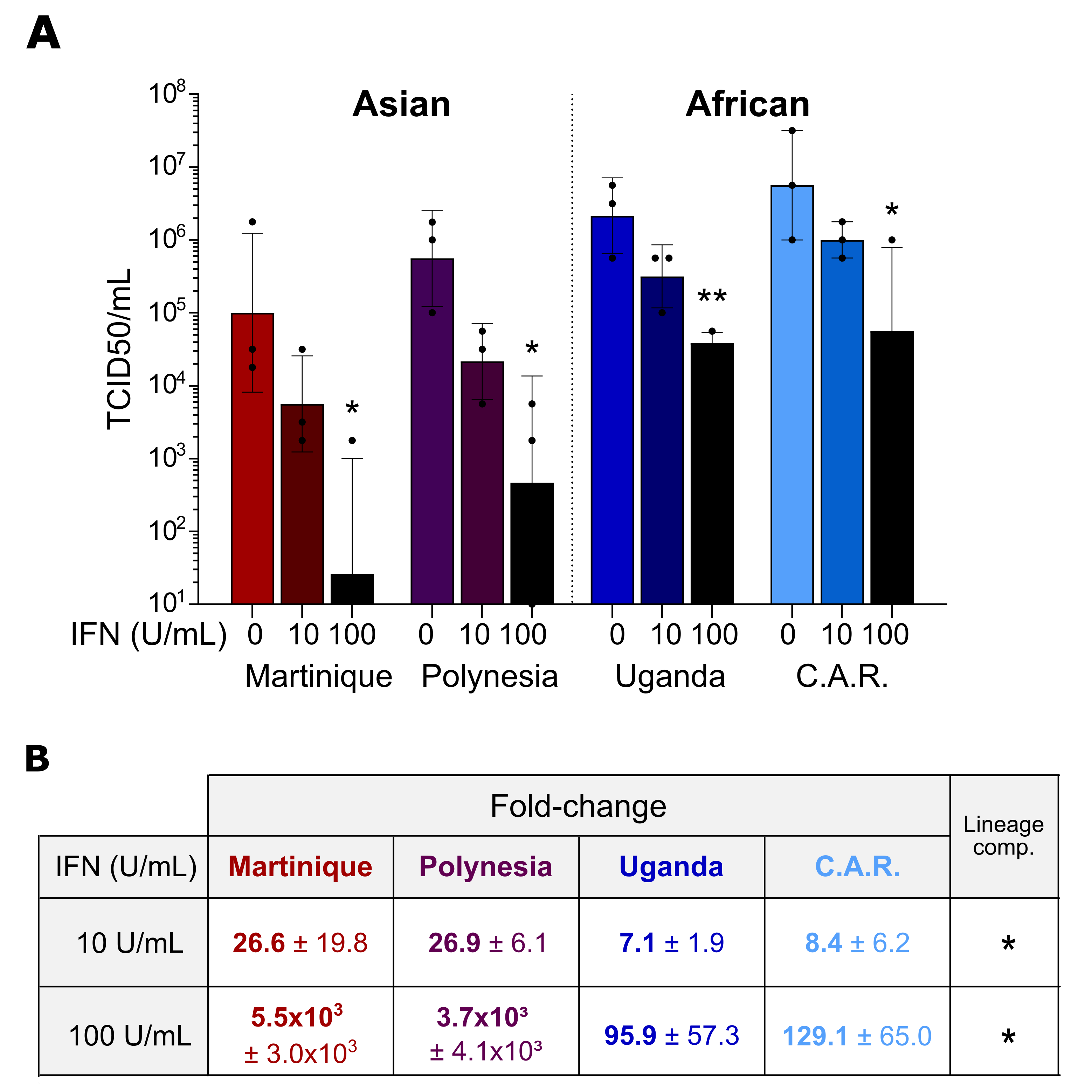

Supplement: S1 Fig — Human monocytes were isolated from peripheral blood mononuclear cells (PBMCs) by plastic adhesion. Following isolation, cells were stained for CD3 and CD14 to assess purity and analyzed by flow cytometry. Representative dot plots from a typical experiment are shown. (TIF) [file pntd.0013326.s001.tif]

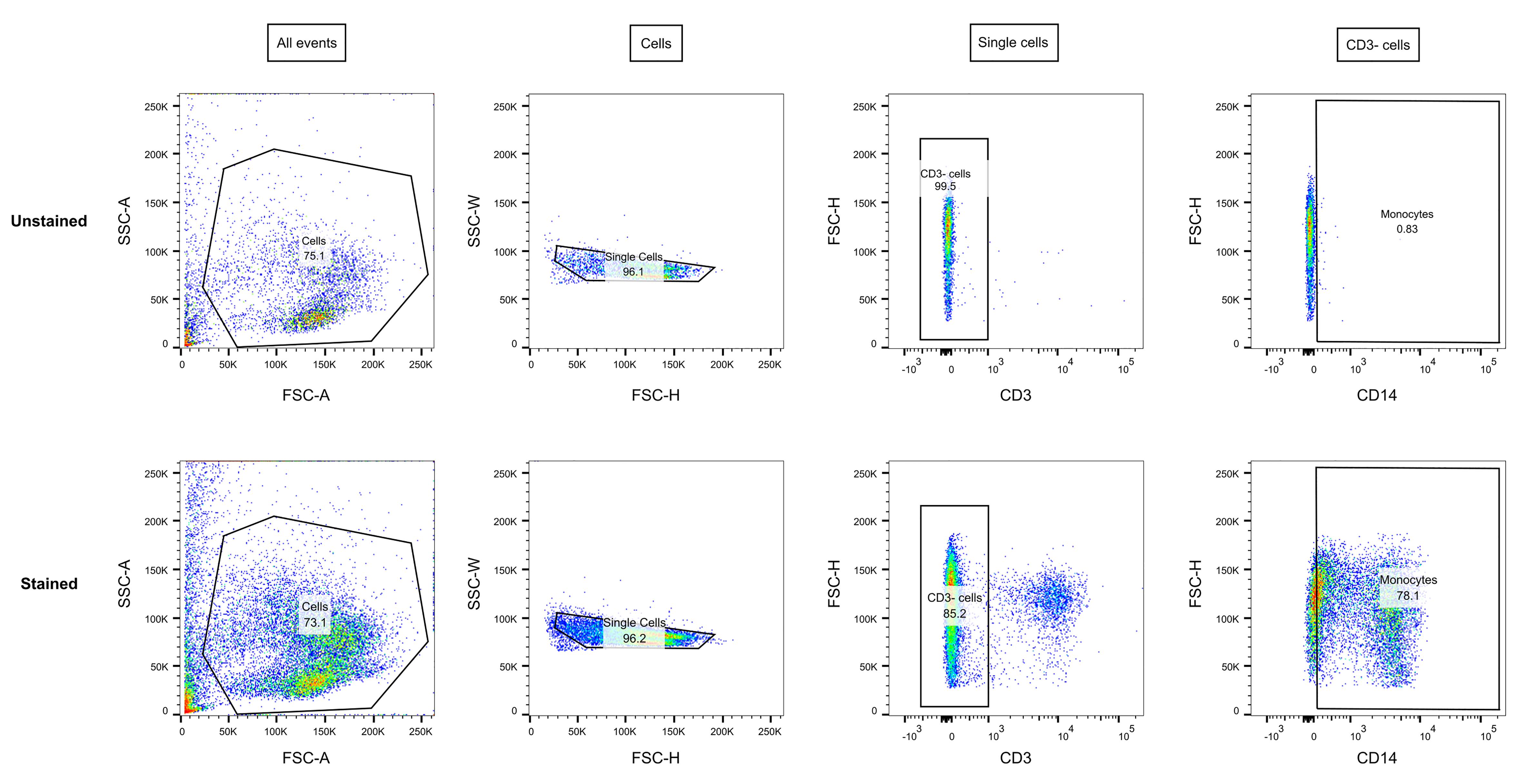

Supplement: S2 Fig — (A) Susceptibility of ZIKV strains to IFN-I was assessed by measuring viral titers in Vero E6 cells. Cells were pre-treated with IFN-I (0, 10, or 100 U/mL) for 16 hours, followed by infection with different ZIKV strains (MOI 0.1). Supernatants were collected at 48 h post-infection, and viral titers were determined by TCID₅₀ assay on Vero cells. Data are shown as individual replicates with mean ± SD (N = 3). Statistical comparisons were made between IFN-treated and untreated conditions for each strain independently using one-way ANOVA with Tukey’s multiple comparisons test. (B) Fold-change in viral titers from panel A, normalized to untreated controls. Data are presented as mean ± SD (N = 3). Statistical comparison between Asian and African strains was performed using a Mann–Whitney test. Statistical significance: *, p < 0.05; **, p < 0.01. (TIF) [file pntd.0013326.s002.tif]

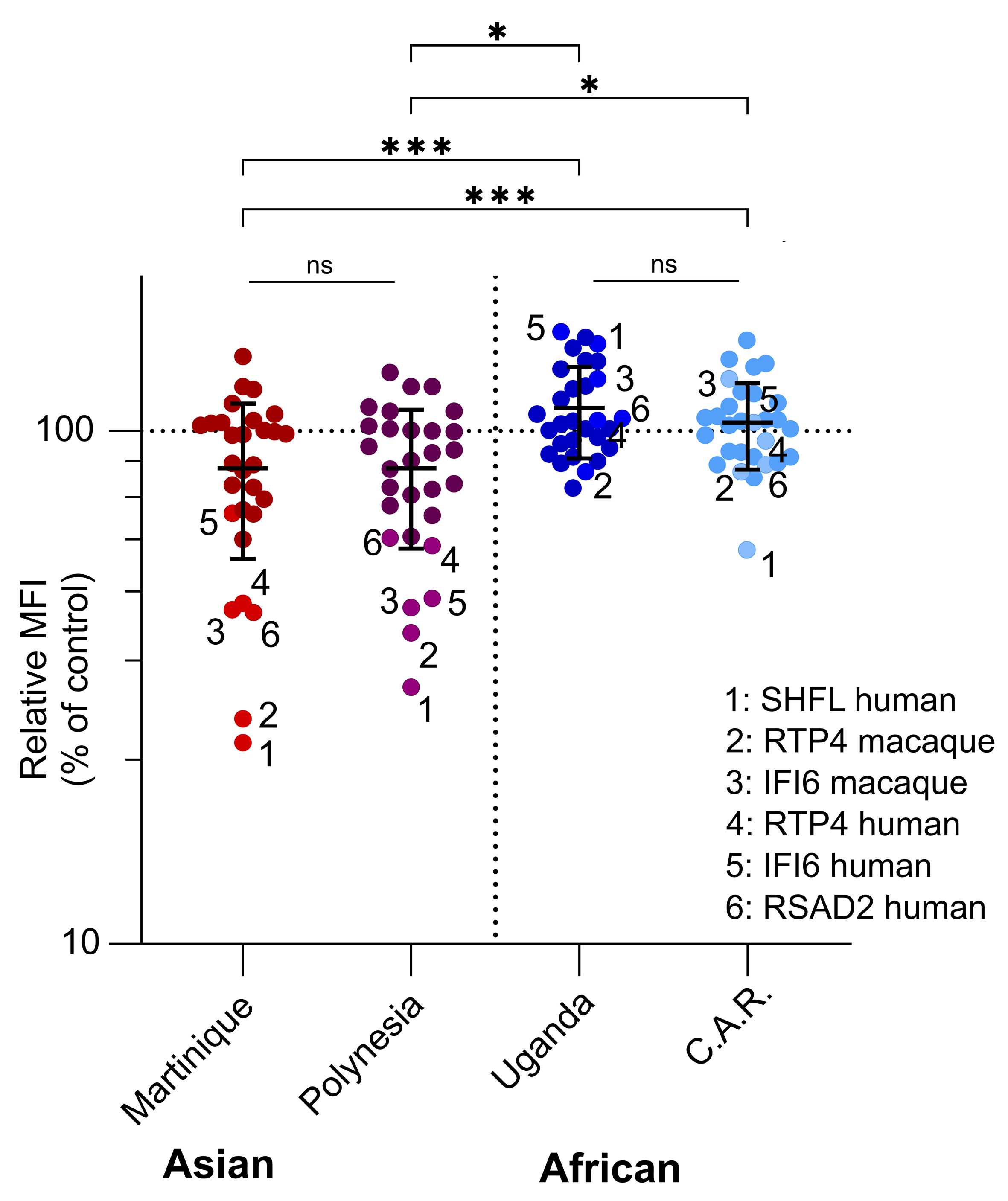

Supplement: S3 Fig — Vero E6 cells were transduced with individual interferon-stimulated genes (ISGs) and subsequently infected with ZIKV. Infection was quantified by flow cytometry based on mean fluorescence intensity (MFI), expressed relative to cells transduced with an empty vector control (set to 100%). Each data point represents the mean of three biological replicates per ISG. Data are shown as individual ISGs with mean ± SD. Statistical analysis was performed using one-way ANOVA followed by Tukey’s multiple comparisons test. Statistical significance: ns, p > 0.05; *, p < 0.05; **, p < 0.01; ***, p < 0.001. (TIF) [file pntd.0013326.s003.tif]
